# Re-Evaluating Recommended Optimal Sleep Duration: A Perspective on Sleep Literacy

**DOI:** 10.3390/children11091098

**Published:** 2024-09-07

**Authors:** Jun Kohyama

**Affiliations:** Tokyo Bay Urayasu Ichikawa Medical Center, Urayasu 279-0001, Japan; info@j-kohyama.jp

**Keywords:** catch-up sleep, ICD-11, ICSD-3-TR, non-communicable disease, sleep inertia

## Abstract

A significant number of adolescents experience sleepiness, primarily due to sleep deprivation. The detrimental effects of inadequate sleep on both physical and mental health are well documented, particularly during adolescence—a critical developmental stage that has far-reaching implications for later life outcomes. The International Classification of Diseases 11th Revision recently introduced the disorder termed ‘insufficient sleep syndrome,’ characterized by a persistent reduction in sleep quantity. However, diagnosing this condition based solely on sleep duration is challenging due to significant individual variation in what constitutes optimal sleep. Despite this, managing sleep debt remains difficult without a clear understanding of individual optimal sleep needs. This review aims to reassess recommended sleep durations, with a focus on enhancing sleep literacy. Beginning with an exploration of insufficient sleep syndrome, this review delves into research on optimal sleep duration and examines foundational studies on sleep debt’s impact on the developing brain. Finally, it addresses the challenges inherent in sleep education programs from the perspective of sleep literacy. By doing so, this review seeks to contribute to a deeper understanding of the chronic sleep debt issues faced by adolescents, particularly those affected by insufficient sleep syndrome.

## 1. Introduction

Sleep accounts for approximately 30% of a human lifetime and is recognized as a critical process for survival [1]. The assessment of sleep necessitates the consideration of both quality and quantity [2]. Numerous factors influencing sleep quality have been identified [3], and the interrelationships between sleep quality, metabolic syndrome, and heart rate variability have been explored [4]. However, the correlation between sleep quality and physiological indicators remains weak [5]. The majority of studies assessing sleep quality rely on subjective measures [4,6], and it has recently been highlighted that the correlation between subjective sleep quality and objective sleep metrics is relatively weak [7]. Furthermore, the research on the adverse effects of poor sleep quality is less extensive compared to studies examining the impact of reduced sleep quantity on physical and mental health. Although recommendations for sleep quantity exist [8,9] (Table 1), there are no specific guidelines for sleep quality.

In the “Sleep Guide for Health Promotion 2023 in Japan,” recommendations for appropriate sleep duration and ensuring a reduction in “nonrestorative sleep” are proposed according to age groups, equating “nonrestorative sleep” with sleep quality [10]. Notably, a decrease in “nonrestorative sleep” is strongly associated with a decline in self-perceived health [11] and has been linked to the onset of depression [12], as well as to the severity of depressive symptoms [13]. Factors contributing to a decline in “nonrestorative sleep” include chronic conditions such as diabetes, hypertension, cancer, and depression [14], although no global recommendations for “nonrestorative sleep” have been established. Poor-quality sleep significantly impacts various aspects of adolescent health and daytime functioning, resulting in diminished cognitive abilities (such as inattention and reduced concentration), lower academic performance, impaired emotional regulation, and heightened behavioral and psychopathological issues [15]. Moreover, during adolescence, there is an increased preference for eveningness [16], along with changes in the hormonal environment [17], both of which further influence sleep quantity and quality. 

**Figure 1:**
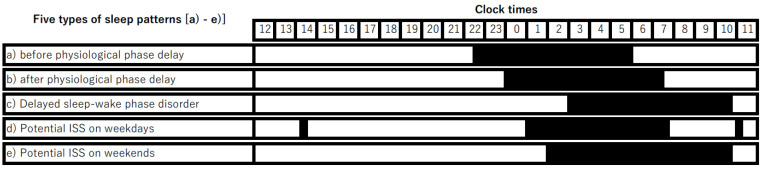
Schematic drawing of the five types of sleep patterns. This figure presents a schematic representation of five types of sleep patterns (a–e), with the horizontal axis representing the 24 h period from noon to the following noon. The periods of sleep are indicated in black. (a) A pupil before the physiological phase delay, who went to bed at 22:30 and woke up at 6:00, resulting in a total sleep duration of 7.5 h. (b) A pupil after the physiological phase delay, who went to bed at 0:00 and woke up at 7:30, also with a total sleep duration of 7.5 h. (c) A pupil with delayed sleep–wake phase disorder, who went to bed at 3:00 and woke up at 10:30, with a total sleep duration of 7.5 h. (d) A pupil with potential insufficient sleep syndrome (ISS) on weekdays, who went to bed at 1:00 and woke up at 7:30, taking 15 min naps during morning and afternoon school classes, with a total sleep duration of 7.0 h on weekdays. (e) A pupil with potential ISS on weekends, who went to bed at 2:00 and woke up at 10:30, resulting in a total sleep duration of 8.5 h on weekends, including 1.5 h of catch-up sleep. The weekly sleep duration of a pupil with potential ISS who exhibited patterns (d) and (e) was 52.0 h, which is 0.5 h shorter than that of pupils exhibiting patterns (a), (b), and (c). Persistent adherence to this sleep pattern may lead to an accumulation of sleep debt, thereby exacerbating difficulties with morning wakefulness.

Recently, the International Classification of Diseases 11th Revision (ICD-11) [18] has recognized a disorder known as insufficient sleep syndrome (ISS), characterized by reduced sleep quantity. This disorder has also been acknowledged in the *International Classification of Sleep Disorders, Third Edition, Text Revision* (ICSD-3-TR) [19]. Figure 1 schematically illustrates the sleep patterns commonly observed during adolescence, including an increased preference for eveningness, delayed sleep–wake phase disorder, and ISS. This review will primarily focus on ISS and begins with an overview of ISS, with a particular emphasis on its prevalence and impact during adolescence, followed by a discussion on the research on optimal sleep duration (OSD). Addressing sleep debt without understanding one’s OSD is challenging [20]. Finally, this review examines the basic research on sleep debt in the developing brain and discusses future directions, especially in the context of sleep education programs.

## 2. Insufficient Sleep Syndrome (ISS)

### 2.1. Diagnostic Criteria

Between 25% and 84% of adolescents report experiencing sleepiness, with sleep deprivation identified as the primary cause in this age group [21]. While a short sleep duration induces drowsiness, there is considerable individual variability in the required amount of sleep, or OSD, making it impossible to diagnose ISS based solely on sleep duration [22]. The diagnostic criteria for ISS, as outlined in the ICSD-3-TR, are presented in Table 2 [19]. Although ISS can affect individuals across all age groups and sexes, it is more frequently observed during adolescence [19].

However, there has been variability in the diagnostic criteria for ISS. Mader et al. [23] and Kohyama et al. [24] used the ICSD-3 criteria, while Jeon et al. [25] and Pallesen et al. [26] employed the ICSD-2 criteria. Both sets of criteria did not specify sleep duration requirements. In contrast, Lee et al. [27,28] defined ISS using criteria of short sleep duration on weekdays (≤7 h) and prolonged weekend oversleep (≥2 h), while Morita et al. [29] utilized similar criteria for sleep duration. Kayaba et al. [30] defined ISS based on a weekday sleep duration of <6 h and an extension of sleep duration on weekends ≥ 2 h. Williams et al. [31] defined ISS as insufficient sleep (≤6.5 h). This variability underscores the necessity of specific criteria in research.

### 2.2. Features

During adolescence, the high demand for sleep is often compromised by social and academic pressures, which encourage late-night wakefulness and contribute to a restricted sleep duration [19]. Additionally, physiological tendencies toward a delayed sleep phase [16] and a preference for later bedtimes further exacerbate chronic sleep restriction in this population [19]. The primary symptom of ISS is drowsiness; however, individuals with ISS may not always recognize sleep deprivation as the underlying cause of their drowsiness [23]. While prolonged sleep deprivation is known to impair performance, the severity of drowsiness in sleep-deprived persons does not always correspond to the extent of sleep loss [32]. Similarly, some patients with ISS may not identify their drowsiness but instead report symptoms such as irritability, inattention, dysphoria, decreased cooperativeness, lack of motivation, fatigue, lassitude, loss of appetite, and muscle pain [23].

ISS may also impact autonomic nervous system activity. Among 102 Korean students with a mean age of 17.1 years, the snoring scores of non-ISS students (n = 52) showed no significant correlation with any heart rate variability-related indices [25]. In contrast, the snoring scores of ISS students (n = 50) demonstrated a significant positive correlation with the total power of heart rate variability and the standard deviation of the normal-to-normal interval, as well as a significant negative correlation with approximate entropy, a measure of the regularity and complexity of a time series [25].

### 2.3. Prevalence

In a study of 1285 Norwegian high school students aged 16–19, 10.4% were identified as having ISS [26]. Factors associated with ISS included alcohol consumption, urban residency, poor academic performance, anxiety, and depression [26]. Similarly, in a study involving 8010 South Korean students in grades 7–11 (mean age, 16.7 years), 18.8% were found to have ISS, with the ISS group scoring significantly higher on a suicide ideation scale compared to the non-ISS group [27]. Additionally, from the same cohort, 51 ISS students and 53 non-ISS students were randomly selected for further analysis. The ISS group exhibited significantly poorer academic performance, a tendency towards eveningness, depressive symptoms, and impulsivity compared to their non-ISS counterparts [28]. In the United States, 98 out of 989 university students (10%) were diagnosed with ISS, and a significant association between ISS and depression was reported [31].

In Japan, Kayaba and colleagues examined 1034 university students and found that 74 had ISS, highlighting a correlation between ISS and negative impacts on academic performance and punctuality [30]. Morita and colleagues reported that 10.9% of 2276 individuals aged 20–25 had ISS, with a higher prevalence among students and full-time employees, and noted a relationship between ISS and both depression and poor health-related quality of life [29].

Among 181 patients under the age of 20 who visited the author’s sleep clinic, ISS was the most common final diagnosis, affecting 56 patients [24]. The primary complaints among these patients were difficulty waking up in the morning (35 patients), daytime drowsiness (20 patients), and a sick feeling in the morning (1 patient). As part of cognitive behavioral therapy, all patients were requested to keep a sleep diary, with discussions of diary entries emphasized during clinic visits. The main principle of treatment is to ensure adequate sleep time, which can be challenging to achieve. When improvement is observed, patients often report statements such as, “If I sleep for more than XX hours, I can stay awake at school,” or “I feel refreshed if I go to bed before XX o’clock.” These statements correspond to Criterion E of the diagnostic criteria (Table 2). It is essential to support adolescents who are struggling to balance basic social demands with their biological needs [33].

### 2.4. Catch-Up Sleep

The average weekday sleep duration just before symptom onset for the aforementioned 56 ISS patients [24] was 7.4 h for those aged 9–15 and 6.1 h for those aged 16–20, both shorter than the recommended sleep duration (9–11 h for ages 6–13, 8–10 h for ages 14–17, and 7–9 h for ages 18–25) [8]. On weekends, the average sleep duration for these patients was 10.7 h for those aged 9–15 and 9.9 h for those aged 16–20. The difference between average weekend and weekday sleep durations was 3.3 h for those aged 9–15 and 3.8 h for those aged 16–20. In contrast, the difference between weekend and weekday sleep durations for Japanese teenagers in 2020 was 1.3 h [34], indicating that ISS patients experience a difference more than two hours longer than this average. Adolescents often do not obtain their OSD on weekdays and compensate for this sleep deprivation by sleeping longer on weekends [35]. The awareness of sleep deprivation among adolescents is typically low at the beginning of the week but increases towards the end of the week [36], suggesting that longer sleep on weekends helps mitigate their perceived sleep debt [37]. As early as 1983, it was noted that ISS patients exhibited longer sleep durations on weekends compared to weekdays [38]. The difference in sleep duration between weekends (or holiday/non-school days) and weekdays (school days) is referred to as weekend catch-up sleep (CUS) or weekend oversleep, which is considered an indicator of sleep deprivation [39] (Figure 1d,e). According to ICSD-3-TR [19], it is also noted that ISS patients sleep longer on weekends or during vacations.

### 2.5. Recent Developments

The following description was added to the ISS section of ICSD-3-TR [19]: difficulty awakening in the morning (sleep inertia) is particularly evident in adolescents. Prolonged and severe sleep inertia, referred to as sleep drunkenness, describes the symptoms observed when attempting to forcibly wake a patient who cannot awaken in the morning. Specifically, this state of confusion includes unresponsiveness, intense resistance or aggressive behavior, and verbal violence that the individual does not later remember. Although this condition was initially considered a core symptom of idiopathic hypersomnia, it is now recognized as also occuring in other hypersomnias and in delayed sleep–wake phase disorder [40].

Recently, it has been reported that Criterion E of the ICSD-3-TR diagnostic standards is not necessarily confirmed in many suspected cases of ISS, raising questions about the validity of the current diagnostic criteria [41]. However, the negative impact of sleep deprivation on both physical and mental health is well established, and there is significant concern regarding the effects of sleep deprivation during developmental stages, particularly adolescence, on later life. With the inclusion of ISS in ICD-11 [18], it is hoped that this condition will gain broader recognition as a disorder, leading to advancements in research, including a review of the diagnostic criteria.

## 3. Optimal Sleep Duration

### 3.1. Optimal Sleep Duration and Habitual Sleep Duration

The study by Roffwarg et al. [42] is widely recognized for its analysis of age-related changes in sleep duration, demonstrating that total sleep time gradually decreases from infancy to old age. The reported values for different age groups are as follows: 10.5 h for ages 5–9, 10 h for ages 10–13, 8.5 h for ages 14–18, and 7.75 h for ages 19–30. Similarly, Iglowstein et al. [43] observed a reduction in sleep duration from early to late adolescence, with sleep durations of 9.9 h at age 10, 9.0 h at age 13, and 8.1 h at age 16. However, these values should be interpreted as the habitual sleep duration (HSD) in the context of typical daily life, rather than as the biologically necessary optimal sleep duration (OSD). When a significant discrepancy exists between OSD and HSD, the need for CUS inevitably increases. If this sleep deficit is not adequately addressed, it may contribute to the development of ISS. Therefore, it is imperative to implement urgent measures to address sleep deprivation among adolescents, regardless of whether they have ISS or are at risk of developing it.

### 3.2. Recommendations for Sleep Duration

In light of these considerations, what reference should adolescents experiencing sleep deprivation, whether or not they have ISS, use to determine their own sleep duration? A crucial metric in this context is the recommended sleep duration for different age groups, which is publicly available. Table 1 presents two such recommendations [8,9]. However, the required amount of sleep can vary significantly among individuals, making it essential to determine one’s own necessary sleep duration. The author suggests considering the following three indicators of insufficient sleep duration: (1) excessive oversleeping on days off, (2) morning drowsiness, and (3) exceptionally rapid sleep onset. Indicator (1) reflects a high level of accumulated sleep debt (CUS), as previously discussed. With regard to (2), it is known that the amount of slow-wave sleep peaks twice daily, at midnight and in the afternoon [44]. This means that even in the absence of sleep deprivation, individuals may feel drowsy at midnight and in the afternoon. However, as diurnal animals, humans should generally not experience drowsiness in the morning. As for (3), while some individuals may boast about falling asleep within five seconds of going to bed, such behavior is a clear indication of sleep deprivation. If any of these three indicators apply, it suggests that one’s sleep duration is insufficient. It is necessary to increase sleep time to a level where these symptoms no longer occur. Although no definitive guidelines exist for this recommendation, continuous efforts to provide indicators for determining each individual’s required sleep duration (OSD) are essential.

### 3.3. Methods of Estimating OSD 

Traditionally, OSD is determined by allowing participants to sleep for extended periods over multiple nights, ensuring that they do not accumulate sleep debt. As their sleep duration gradually stabilizes, the consistent length is considered the OSD. Carskadon et al. [45] conducted a study involving 47 sleep tests on 19 children (11 boys and 9 girls, aged 10 to 17), analyzing the results based on sexual maturity. Participants underwent night-time sleep tests with a 10 h bed rest period (from 10 p.m. to 8 a.m.), during which OSD was measured. The average total sleep time was 9.0 h (standard deviation: 33 min) for Tanner stage 1, 9.1 h (standard deviation: 25 min) for stage 2, 9.2 h (standard deviation: 16 min) for stage 3, 9.1 h (standard deviation: 30 min) for stage 4, and 8.9 hours (standard deviation: 26 min) for stage 5, showing no significant differences based on sexual maturity.

Wehr et al. [46] reported that in a study with 15 healthy young adults aged 20–36 years, the total sleep duration was significantly longer during nights, with a 10 h daily photoperiod (mean: 8.23 hours; standard deviation: 0.85 h) compared to nights with a 16 h daily photoperiod (mean: 7.18 h; standard deviation: 0.26 h). According to Dement [47], Barbato et al. [48] monitored the sleep of eight healthy males with an average age of 29.0 years over five weeks. The daily photoperiod was set at 16 h in the first week and was reduced to 10 h over the following four weeks. Participants were instructed to remain in bed and sleep as much as possible during the dark period. The average night-time sleep duration in the first week was 7.6 h. When the dark period increased to 14 h in the second experimental week, their average total sleep time exceeded 12 h on the first night and gradually decreased to an average of 8.25 h by the fourth experimental week (the third week of the longer-dark-period session). The authors concluded that the OSD for these eight participants was 8.25 h.

Klerman and Dijk [49] conducted an experiment in which participants were required to remain in bed for 16 h per day over a period of 3 to 7 days. Initially, the total daily sleep duration exceeded the HSD. However, during the experiment, the average sleep duration decreased to 8.9 h (standard deviation: 2.4 h) in younger participants (18–30 years old) and 7.4 h (standard deviation: 1.7 h) in older participants (60–80 years old). Short et al. [50] reported an OSD of 9.1 h (range: 8.5 to 9.5 h) in a study of 34 individuals aged 15 to 17, achieving under 10 h of enforced bed rest during the night. Kitamura et al. [51] extended the bed rest period to 12 h for 9 days in an experiment with 15 healthy adult males, with an average age of 23, whose HSD averaged 7.4 h based on a 2-week home record. They found that the sleep duration on the first day exceeded 10 h but gradually stabilized at an average of 8.4 h (range: 7.3 to 9.3 h) from the fourth day onward, concluding that 8.4 h represented the average OSD for these individuals.

Van Dongen et al. [32] and Short et al. [50] employed a psychomotor vigilance task to estimate the maximum length of daily wakefulness that would not result in deficits in sustained attention, thereby estimating the necessary night-time sleep duration—OSD. Their estimates were 15.84 h for adults and 14.65 h for adolescents, leading them to conclude that 8.16 h [32] and 9.35 h [50] of sleep are required to maintain an optimal sustained attention performance. This estimation may be considered equivalent to OSD.

In a study involving 1689 individuals aged 10 to 18, the sleep duration associated with the highest scores on four different test items was determined to be 8.3 to 8.4 h at age 12 and 7.0 to 7.4 h at age 16 [52]. The authors referred to this sleep duration as the “optimal” sleep duration. Additionally, another study by Fuligni et al. [53] found that the OSD for academic performance among 421 individuals with an average age of 15.0 years was 7.0 to 7.5 h, while the OSD necessary to maintain mental health was 8.8 to 9.0 h. They concluded that sleep duration presents a trade-off between academic performance and mental health [53]. However, it is questionable whether students with the OSD consistently exhibit high academic performance. Although sleep debt may be associated with poor academic performance, other factors such as individual capabilities may also play a significant role. Indeed, sleep duration has shown a weaker correlation to academic performance than to sleepiness [54,55,56].

A formula to estimate OSD in adolescents has recently been proposed [57]. This formula emphasizes body mass index (BMI) and self-reported sleepiness. Given the U-shaped relationship between BMI and sleep duration [58,59,60], it is anticipated that individuals with an OSD close to the population mean will not exhibit significant deviations from the average BMI. In this model, students who reported never feeling sleepy during class (selecting “never” from the following options: 1. never, 2. sometimes, 3. often, 4. always) and who had a gender- and grade-standardized BMI within ±1.5 were classified as ‘ideal students.’ The difference in average sleep duration between ideal and non-ideal student groups was added to the HSD of each non-ideal student to calculate the “assumed OSD.” Subsequently, using the least-squares method, a multiple regression line was calculated to predict the “assumed OSD,” yielding the following significant regression formula: 0.714 × (sleep duration before schooldays) + 0.284 × (sleep duration before non-schooldays) + 0.513 × (sleepiness score; 1–4) + 0.002 × (grade; 5–11) − 0.009 × [gender (male:1; female: 2)] − 0.005 × [social jetlag (hours)] + 0.008 × (standardized BMI) − 0.501. Recognizing one’s OSD is critical in reducing sleep debt [20], and this formula may serve as a valuable tool for aiding sleep-deprived adolescents. This equation indicates that greater sleepiness and a higher BMI are associated with a need for a longer sleep duration than the current sleep duration, whereas a larger social jetlag is associated with a shorter required sleep duration compared to the current one. 

Regarding chronological changes and gender differences in OSD, this formula highlights important aspects. According to the formula, an increase of one grade level is associated with a prolonged daily OSD of 0.002 h (0.12 min). However, this finding contrasts with previous research, which reported no chronological changes in OSD during adolescence [45,50]. Moreover, recent studies have shown that night-time sleep duration for individuals aged 5–18 years decreased by an average of 0.75 min per year between 1905 and 2008 [61]. This well-documented reduction in HSD during adolescence [42,43] supports the hypothesis that the decline in sleep duration is more reflective of social influences, such as increased light exposure, rather than a biological reduction in the need for sleep, or OSD.

Regarding gender differences, Dietch et al. [62] reported that adult females aged 21 to 70 years tend to sleep more than their male counterparts, whereas Jiang et al. [63] found the opposite pattern among children aged 8 to 16 years. According to the proposed formula, females have a longer daily OSD by 0.009 h (0.54 min) than males. Further research is necessary to draw definitive conclusions on this matter.

Several limitations were noted in deriving this equation [57]. First, the questionnaire used was neither standardized nor validated. Second, the study relied on self-reported data from students, lacking direct measurements of sleep duration, height, weight, and other assessed variables. Third, the sleepiness scale was based on a single question with four response options, rather than utilizing a standardized scale. Fourth, demographic factors were not measured in this study. Finally, the cross-sectional design of this study poses limitations in establishing causality.

### 3.4. Recent Topics

Recommending an OSD for the general population has long been a critical objective in public health, as both short and long sleep durations have been associated with adverse health outcomes [64]. A recent study has emphasized that deviations from OSD—whether insufficient or excessive—have a particularly pronounced impact on mental health and quality of life during adolescence compared to other age groups [65]. Therefore, it is crucial for adolescents to be more attuned to their own OSD than individuals in other age groups. From this perspective, the existence of a straightforward formula to estimate the OSD for adolescents [57] would be of significant importance. Furthermore, adverse childhood experiences before the age of 18 have been shown to increase the likelihood of developing chronic short sleep duration in adulthood, subsequently reducing OSD later in life [66]. Sleep duration is subject to various influences, including seasonal variations [67], lunar cycles [68], and ambient temperature [69]. Consequently, OSD may also fluctuate in accordance with circadian factors. Furthermore, van de Langenberg et al. [64] argue that sleep is not merely a function of duration but a complex physiological process that must be evaluated across multiple dimensions, including timing, regularity, satisfaction, alertness, and efficiency. In fact, sleep regularity has been recognized as a more significant predictor of mortality risk than sleep duration [70]. While substantial interindividual variation in OSD is well-documented, it is also suggested that HSD within the optimal range may be partly regulated by genetic factors related to the immune system [71].

## 4. Causes of Insufficient Sleep

Inadequate sleep in adolescence is caused by factors such as a shift towards eveningness during puberty [16], a slower “sleep drive,” [72] and external factors like extracurricular activities [72], heavy homework loads [72], evening use of electronic media [72,73], and caffeine intake [72].

Although it remains unclear whether COVID-19 infection itself or subsequent factors such as school closures, online learning, and changes in social support directly impact sleep, Hossain et al. [74] highlighted that the pandemic has led to a wide range of mental health issues in children and adolescents, with sleep disorders being a significant concern. Panchal et al. [75] reported a prevalence of 55.9% for sleep disorders among adolescents. According to Falkingham et al. [76], the pandemic has increased sleep deprivation, especially among women with young children, people infected with COVID-19, and individuals from Black, Asian, and Minority Ethnic communities. They also noted that this sleep loss may harm their mental and physical health [76].

## 5. Potential Biological Consequences of Insufficient Sleep

Insufficient sleep can lead to diminished cognitive and emotional functioning [77]. Consequences include fatigue [73], daytime sleepiness [73], inattention, reduced executive functioning, and poor academic performance [72]. Additionally, it is associated with an increased risk of obesity, cardiovascular and metabolic dysfunction, mood disturbances such as heightened suicidal ideation, and a greater likelihood of engaging in health risk behaviors like alcohol and substance use [72]. Moreover, insufficient sleep is linked to higher rates of car crashes, occupational injuries, and sports-related injuries [72] and may have long-term adverse effects on cardiovascular and metabolic systems [15]. In addition, a shorter sleep duration has been linked to heightened inflammatory responses and an elevated risk of infection [78]. 

## 6. Basic Data on the Impact of Sleep Deprivation on the Developing Brain

It has become increasingly evident that chronic sleep deprivation has a profound impact on brain health and function [35]. In young mice, chronic sleep deprivation leads to increased vulnerability to neuronal degeneration among those neurons that are active during the waking period. Additionally, it is now recognized that chronic sleep deprivation affects not only neurons but also oligodendrocytes, astrocytes, and microglia. The molecular mechanisms underlying these effects are beginning to be elucidated. In rats subjected to chronic sleep deprivation, potential biomarkers have been identified [79], and apoptosis, along with a reduction in the number of neurons and glial cells, has been observed in the superior cervical ganglion [80].

Gay et al. [81] conducted a systematic examination of the behavioral and molecular responses to acute sleep deprivation in juvenile (P21–28), adolescent (P42–49), and adult (P70–100) mice of both sexes. Their findings revealed that juvenile mice exhibit inadequate adaptations to sleep deprivation compared to adults, resulting in cognitive deficits as measured by the novel object recognition test.

In a study involving mice, a 14-day period of sleep deprivation starting on postnatal day 28 (PND28) resulted in significant impairments in long-term memory by PND42 [82]. Notably, these impairments persisted into adulthood at PND85. Histopathological changes in hippocampal neurogenesis, as indicated by bromodeoxyuridine labeling, were observed and are believed to contribute to the long-term memory deficits at both PND42 and PND85. Additionally, the hippocampal region displayed significantly reduced protein expression of astrocytes, characterized by decreased levels of aquaporin 4—a key molecule involved in brain clearance processes—and diminished protein expression of brain-derived neurotrophic factor.

In adolescent mice, there was a 2% net loss of dendritic spines following sleep and a 1% gain following wakefulness, whereas the spine turnover in the sensorimotor cortex was unaffected by sleep and wakefulness in adult mice. This suggests that spine turnover in the sensorimotor cortex is particularly sensitive to behavioral states during early adolescence [83].

Maric et al. [84] investigated risk-seeking behavior and electroencephalographic findings in 14 right-handed male students aged 18–28 years (mean age 21.9 years) with an average sleep duration of 7.7 h. They compared chronic sleep deprivation, characterized by a restriction of sleep to 5 h per night for one week, with acute sleep deprivation, involving 40 consecutive hours of wakefulness. The study found that risk-seeking behavior increased solely under chronic sleep deprivation, accompanied by a decrease in slow-wave activity in the right prefrontal cortex. Notably, slow-wave activity is indicative of sleep recovery. Interestingly, participants did not perceive the increase in risk-seeking behavior. These findings suggest that chronic sleep deprivation adversely affects decision-making.

Although conducted on healthy adults, the experiment demonstrated that sleep deprivation impacts vigilance by altering electroencephalographic spectral power and information transmission across frequency bands in different brain regions [85]. Increased theta power and delta/theta-gamma phase-amplitude coupling may, respectively, reflect the impairment and compensation of vigilance due to sleep deprivation.

In adolescents with shorter sleep durations, elevated TNF levels were associated with reduced activation in the medial frontolimbic circuitry compared to those with longer sleep durations, and the degree of deactivation correlated with increased self-reported anxiety [86]. Regarding brain connectivity, common resting-state functional brain connectivity patterns are reported to reflect sleep duration in youth and young adults [87]. Guldner et al. [88] reported that insufficient sleep in adolescents could have potentially detrimental effects on long-term white-matter development, as measured by diffusion tensor imaging and estimated using fractional anisotropy. They also noted that longer sleep durations on weekends (catch-up sleep) might serve as a natural countermeasure and protective strategy.

Oxidative stress and sustained inflammatory responses are potential mechanisms underlying brain damage due to chronic sleep deprivation [89]. Additionally, although still hypothetical, the glymphatic system—thought to be activated during sleep to clear brain waste—represents an intriguing area of ongoing research [90].

## 7. Sleep Education for Adolescence with Sleep Debt

Adolescents experiencing chronic sleep deprivation or diagnosed with ISS face a range of challenges [91]. These challenges include persistent daytime drowsiness, academic difficulties, health issues such as obesity, mental health concerns like depression, engagement in risky behaviors (e.g., alcohol consumption, smoking, and drug use), and safety risks, including an increased likelihood of traffic accidents. To ensure adequate sleep, it is crucial to establish a consistent wake-up time, limit the use of electronic devices before bedtime, and engage in regular physical activity [92]. School administrators should seriously consider delaying school start times to facilitate sufficient sleep for adolescents, and policymakers need to develop strategies to enhance public awareness regarding the importance of adequate sleep [15,91]. Van Rijn et al. [93] emphasize that altering adolescent sleep habits requires not only sleep education but also social and familial involvement. While improving sleep hygiene practices can enhance sleep quality, there remains a gap in understanding the most effective methods for improving sleep hygiene in this population [94]. The application of cognitive behavioral therapy techniques, such as the use of sleep diaries, will be increasingly important [95].

A school-based intervention involving a ten-lesson program led by teachers was conducted for 1504 students with a mean age of 14 years. Although there were notable improvements in sleep understanding, there was only a slight improvement in sleep quality and hygiene, with no effect on sleepiness or quality of life [96]. Similar results were observed in a study of 13–14-year-old students in Australia [97].

In Japan, a significant social initiative was the establishment of the “Early to Bed, Early to Rise, and Breakfast” National Council in April 2006 [98]. Reports indicate that sleep guidance for elementary school students has helped reduce absenteeism following their transition to junior high school [99]. Furthermore, community awareness activities related to sleep have been reported to improve sleep habits, morning conditions, and self-esteem among junior high school students [100]. However, globally, there are few reports of effective measures. One study outlines three key approaches for addressing adolescent sleep issues: (1) presenting the benefits of adequate sleep, (2) securing parental support, and (3) exercising patience as effects may take time to manifest. However, this study does not document any clear effects [101]. While cultural differences may influence outcomes, Japan’s methodologies are highly anticipated to provide effective solutions. 

According to Bonuck et al. [102], “sleep health literacy” refers to the knowledge, motivation, and competencies required to promote healthy sleep and recognize sleep problems. Even among healthcare professionals, low sleep literacy can lead to shortcomings in clinical practice [103]. An internet-based educational program has been reported to significantly and persistently improve sleep literacy among college psychology students, not only in terms of sleep knowledge but also particularly in their sleep habits, such as wake times [104]. As previously emphasized, effectively managing sleep debt requires individuals to understand their optimal sleep duration (OSD) [20], which is central to the concept of sleep literacy. In contrast, the currently available recommended sleep durations [8,9] are overly generalized and encompass a broad range, making them inadequate for providing tailored interventions for sleep deprivation and ineffective from the perspective of sleep literacy. Therefore, the introduction of a formula that enables individuals to easily calculate their own OSD [57] holds significant potential to transform future sleep education programs.

As previously mentioned, simply advising sleep-deprived adolescents to “get more sleep” is often ineffective in clinical settings. To facilitate meaningful improvement, it is essential to propose specific, measurable targets that can serve as attainable goals. In this regard, the formula introduced in Section 3.3 [57] is considered useful. For instance, when adolescents report that “getting X hours of sleep makes mornings easier and helps them stay awake in class,” it underscores the importance of providing tailored recommendations that they can personally experience and benefit from. Furthermore, in my sleep clinic, I explain the following seven guidelines [105] (Table 3) as part of the basic recommendations. These guidelines are largely based on the “Good Sleep Guide,” [10] with the addition of recommendations regarding excretory habits, which are derived from my own research findings [105,106]. In a study involving 2722 students from grades 5 through 12, defecation habits were categorized into four groups: “daily,” “every other day,” “once every 2–3 days,” and “less than twice per week.” Comparative analysis of sleep patterns revealed that the group with defecation habits of less than twice per week, meeting the international criteria for constipation [107], had significantly later bedtimes compared to the daily bowel movement group (weekdays: 23:13 vs. 22:46; weekends: 23:28 vs. 23:02). Additionally, their weekday sleep duration was significantly shorter than that of the daily bowel movement group (7.29 h vs. 7.63 h) [105].

## 8. Conclusions

ISS is a chronic condition influenced by a combination of environmental, behavioral, and genetic factors, impacting various aspects of physical and mental health. This characterization aligns with the World Health Organization’s definition of non-communicable diseases, which are chronic conditions arising from a complex interplay of genetic, environmental, physical, and behavioral factors [108]. Consequently, there is a growing argument that ISS should be recognized as a non-communicable disease at both national and international levels [109]. The recently proposed formula, which enables individuals to calculate their own OSD [57], is anticipated to be particularly beneficial for adolescents experiencing sleep deprivation, including those with ISS. The current review aims to contribute to the understanding and management of chronic sleep debt, including ISS, among adolescence.

## Figures and Tables

**Table 1:** Sleep duration recommendations (in hours) for children and adolescents.

	National Sleep Foundation’s Sleep Time Duration Recommendations [8]			A Consensus Statement from the American Academy of Sleep Medicine [9]	
Age	May Be Appropriate	Recommendation	May Be Appropriate	Age	Recommendation
6–13 years	7–8 h≤	9–11 h	≤12 h	6–12 years	9–12 h
14–17 years	7 h≤	8–10 h	≤11 h	13–18 years	8–10 h
18–25 years	6 h≤	7–9 h	≤10–11 h		

**Table 2:** Diagnostic criteria for insufficient sleep syndrome [19].

Criteria A–F must be met:
A. The patient has daily periods of irrepressible need to sleep or daytime lapses into drowsiness or sleep or, in the case of prepubertal children, there is a complaint of behavioral abnormalities attributable to sleepiness.
B. The patient’s sleep time, established by personal or collateral history, sleep log, or actigraphy is usually shorter than expected for their age.
C. The curtailed sleep pattern is present most days for at least three months.
D. The patient curtails sleep time by such measures as an alarm clock or being awakened by another person and generally sleeps longer when such measures are not used, such as on weekends or vacations.
E. An extension to the total sleep time results in the resolution of the symptoms of sleepiness.
F. The symptoms and signs are not better explained by a circadian rhythm sleep–wake disorder or other current sleep disorder, medical disorder, mental disorder, or medication/substance use or withdrawal.

**Table 3:** Seven key points essential to the foundation of sleep health literacy.

1. Exposure to Morning Light: The body’s internal clock, located in the suprachiasmatic nucleus of the brain, operates on a cycle slightly longer than 24 h. Morning light exposure, especially after the lowest body temperature is recorded, shortens this cycle to align with the Earth’s 24 h rhythm.
2. Daytime Activity: (1) Exposure to daylight during the day enhances night-time melatonin production, a hormone responsible for inducing sleep, which begins secretion 14–16 h after waking, once it becomes dark. (2) Blue light during the day increases alertness. (3) Moderate physical activity during the day positively impacts night-time sleep.
3. Resting in Darkness at Night: (1) Melatonin secretion is suppressed in bright environments, even at night. (2) Exposure to light during the night, in contrast to morning light, lengthens the internal clock’s cycle, making it harder to fall asleep. (3) The orange hue of sunset, however, does not increase alertness and instead promotes relaxation.
4. Consuming Breakfast and Avoiding Late-Night Meals: According to the latest findings in chrono-nutrition, establishing a healthy rhythm requires consuming breakfast while avoiding late-night meals.
5. Regular Elimination: Studies indicate that individuals who are suffering from constipation go to bed significantly later and have shorter weekday sleep durations than those with daily elimination.
6. Avoiding Stimulants and Excessive Media Exposure: Stimulants such as caffeine, alcohol, and nicotine, along with excessive media consumption, can impair sleep. Media content stimulates the sympathetic nervous system, making it harder to fall asleep, while light from electronic devices suppresses melatonin and prolongs the internal clock’s cycle, delaying sleep onset. Research indicates that adolescents’ lenses transmit light nearly five times more than those of individuals in their 70s without cataracts, making younger people more sensitive to light’s effects on sleep.
7. Respecting Pre-Sleep Rituals: Establishing a consistent pre-sleep routine is essential for signaling to the body that the environment is safe, which can facilitate a smoother transition into sleep.
